# Making SENSE - Sustained Effort Network for treatment of Status Epilepticus as a multicenter prospective registry

**DOI:** 10.1186/s12883-015-0486-y

**Published:** 2015-11-10

**Authors:** Christoph Kellinghaus, Nicolas Lang, Andrea O. Rossetti, Stephan Rüegg, Christian Tilz, Eugen Trinka, Iris Unterberger, Zeljko Uzelac, Felix Rosenow

**Affiliations:** Department of Neurology, Klinikum Osnabrück, Am Finkenhügel 1, D-49078 Osnabrück, Germany; Department of Neurology, University Hospital Schleswig-Holstein, Campus Kiel, D-24105 Kiel, Germany; Department of Clinical Neurosciences, CHUV and University of Lausanne, CH-1011 Lausann, Switzerland; Department of Neurology, University Hospital Basel, Petersgraben 4, CH-4031 Basel, Switzerland; Department of Neurology, Krankenhaus Barmherzige Brüder Regensburg, Prüfeninger Str. 86, D-93049 Regensburg, Germany; Department of Neurology, Christian Doppler Klinik of Paracelsus Medical University, Ignaz Harrerstarsse 79, A-5020 Salzburg, Austria; Department of Neurology, Innsbruck Medical University, Anichstrasse 35, A-6020 Innsbruck, Austria; Department of Neurology, University Hospital Ulm, Oberer Eselsberg 45, D-89081 Ulm, Germany; Epilepsy Center Hessen – Marburg, Department of Neurology, University Hospitals and Philipps-University Marburg, Rudolf-Bultmann-Strasse 8, D-35039 Marburg, Germany; Centre for cognitive Neuroscience Salzburg, A-5020 Salzburg, Austria

**Keywords:** Status epilepticus, Anticonvulsant, Outcome, AED

## Abstract

**Background:**

Evidence regarding the different treatment options of status epilepticus (SE) in adults is scarce. Large randomized trials cover only one treatment at early stage and suggest the superiority of benzodiazepines over placebo, of intravenous lorazepam over intravenous diazepam or over intravenous phenytoin alone, and of intramuscular midazolam over intravenous lorazepam. However, many patients will not be treated successfully with the first treatment step. A large randomized trial covering the treatment of established status (ESETT) has just been funded recently by the NIH and will not start before 2015, with expected results in 2018; a trial on the treatment of refractory status with general anesthetics was terminated early due to insufficient recruitment. Therefore, a prospective multicenter observational registry was set up; this may help in clinical decision-making until results from randomized trials are available.

**Methods/Design:**

SENSE is a prospective, multicenter registry for patients treated for SE. The primary objective is to document patient characteristics, treatment modalities and in-house outcome of consecutive adults admitted for SE treatment in each of the participating centres and to identify predictors of outcome. Pre-treatment, treatment-related and outcome variables are documented systematically. To allow for meaningful multivariate analysis in the patient subgroups with refractory SE, a cohort size of 1000 patients is targeted.

**Discussion:**

The results of the study will provide information about risks and benefits of specific treatment steps in different patient groups with SE at different points of time. Thus, it will support clinical decision-making and, furthermore, it will be helpful in the planning of treatment trials.

**Trial registration:**

DRKS00000725

## Background

Status epilepticus (SE) is a frequent neurological emergency. There is yet no commonly accepted definition regarding duration and features that distinguish an SE from a ‘normal’ epileptic seizure. The International League Against Epilepsy (ILAE) classification for seizures [1] (Commission on Classification and Terminology of the International League Against Epilepsy 1981) defines SE as a seizure that persists ‘for a sufficient length of time or is repeated frequently enough that recovery between attacks does not occur’. Several authors operationalize the term ‘sufficient length of time’ as 30 min [2]. However, almost all generalized tonic-clonic seizures last less than 5 min, and most focal (complex partial or simple partial) seizures last less than 10 min [3, 4]. In addition, there is evidence suggesting that the magnitude of neuronal damage is correlated to SE duration [5]. Therefore, recent studies have adopted a much shorter time of duration such as ten [6] or even 5 min [7, 8] as critical point of intervention. And a new proposal for definition of SE suggests 5 min for convulsive activity and 10 min for non-convulsive (focal) activity as SE [9].

There are several different classification schemes of SE. Some of them focus on clinical semiology [1, 10] or just distinguish between ‘convulsive’ and ‘non-convulsive’, whereas others classify according to time latency and/or response to treatment [11, 12]. Currently, the distinction between ‘initial SE’ for the first 5–10 min and/or before the first treatment step, ‘established SE’ for the following minutes up to a total duration of about one hour during which the second-line treatment is usually given, ‘refractory SE’ for SE that does not respond to first- and second-line therapy, and ‘super-refractory SE’ for SE recurrence after a course of deep analgo-sedation are preferred [12] when describing and discussing SE treatment. These terms will also be used here.

Evidence for efficacy and safety of different treatment options of SE is scarce. There are two large randomized controlled trials assessing the effect of benzodiazepines for the initial treatment in out-of-hospital situations [7, 8]. These showed the superiority of lorazepam over diazepam and placebo [7], and the equivalence of immediate intramuscular application of midazolam over intravenous application of lorazepam [8]. Another trial found superiority of lorazepam over diazepam [13].

One large multicenter trial addressed the initial in-patient treatment of SE [6], where lorazepam was superior to phenytoin alone, while differences between the other treatment arms (phenobarbital, diazepam/phenytoin, lorazepam) were not significant. In this trial, almost half of the patient had received some form of drug treatment before inclusion into the study. Thus, this study covers treatment of initial as well as established SE. In summary, only four large class I – studies are available for treatment of SE in adults, and only the first treatment step was covered sufficiently to allow sound treatment recommendations. In addition there are some smaller randomized controlled trials comparing phenytoin with valproate [14, 15], lorazepam with levetiracetam [16] or other combinations of different substances. A multicenter randomized trial assessing the effectiveness of barbiturates and propofol in refractory SE had to be interrupted due to insufficient recruitment [17]. In general, these studies are mostly small, monocentric, uncontrolled, have serious methodological flaws, or a combination of these factors applies. Therefore, national and international treatment guidelines for established, refractory and super-refractory SE are based on insufficient evidence, in some instances only on some small retrospective case series [12, 18].

There are several problems that have yet precluded the initiation of a large Class-I trial for treatment of established or refractory SE. Most of them are inherent to studies performed in emergency situations: First, owing to the lack of evidence, it is difficult to assess equipoise of different treatment options. Second, while a blinding (preferably of both the patient and the investigator) to the different treatment options remains the gold standard because biases can be avoided, treatment options for established or refractory SE include various intravenous anticonvulsants with very different infusion modalities, side effects, and application precautions. To maintain blinding, these differences have to be taken into account and require considerable efforts regarding packaging, shipping and storage. In addition, it may be difficult if not impossible to establish a common infusion speed or other treatment modalities, without giving advantage or disadvantage to one of the treatment arms that might distort the results and render transfer of the results into clinical everyday practice impossible. For example, one of the disadvantages of phenytoin is the limitation of infusion speed due to the potentially related adverse effects, such as hypotension or cardiac arrhythmia, while, if used with equal infusion speed to phenytoin, valproate and levetiracetam might loose their potential advantage.

Informed consent (IC) causes other problems. In the United States, there is an exemption for emergency care research if the situation under study is emergent and potentially life-threatening. This exemption could be essential for effective research in emergencies with impaired awareness, in which to obtain the valid IC of the patient might be impossible and there is usually no time to contact a proxy. However, in other countries this exemption may not be possible: In Germany, for example, informed consent can be postponed but not entirely waived in such cases.

In fact, the requirement to obtain IC may be one of the main reasons of difficulties in patient recruitment in studies of established and refractory SE. Other reasons include reservations of the treating team against drug studies, lack of resources to adequately perform inclusion assessment, lack of incentives for the institution to support such a trial, availability of drugs, treatment habits of the doctors in an almost evidence free zone, or a combination of any of these factors.

Another aspect increasingly gaining relevance is the potential delay of drug application after the diagnosis of SE. Duration of delayed initiation of treatment after diagnosis of SE has been recently linked with dismal outcome as this factor could contribute partly to rendering status epilepticus “refractory” [19–21]. It would be interesting to see the results, across several medical centers, regarding the timing of treatment with respect to the onset of status.

Finally, the planning, execution and evaluation of a large drug trial involving institutions in several countries with several hundreds of participants is demanding and requires considerable financial and logistic resources. A sufficiently powered trial that is able to detect an effect size of 15 % on the base of a success rate of 50 % for the worst treatment option needs almost 300 patients per treatment arm [22, 23]. If the recruitment phase is supposed to remain below 3–4 years, more than 50 study sites will be required to obtain an accrual rate of 10–15 patients per month [22]. The costs of a pragmatic open-label design of such a trial in Europe were estimated to be at least 5 million Euro [23]. A comparable double-blinded trial might easily require the double to threefold amount of money. Drug companies hesitate to fund a large SE trial under the concern of implications of high serious adverse events and mortality rates, and also of the fact, that the market for intravenous anticonvulsants is rather small. In Europe, no funding agency was prepared to support a large drug trial. The NIH recently granted a trial comparing IV levetiracetam, IV valproate and IV phenytoin/fosphenytoin in treatment of established SE (ESETT) utilizing an adaptive trial design [22]. Even if ESETT is to start in 2015, results cannot be expected before 2018 or even later. At that time, new substances or treatment strategies might have been discovered that will not be included into the study in question.

To bridge the gap between small series and the necessary large randomized trial, an informal working group of experts in status epilepticus from German-speaking countries (Germany, Austria, Switzerland) developed a prospective multicenter registry for patients treated for SE.

## Methods/design

### Study design

SENSE is a prospective, multicenter, non-randomized, observational registry study of consecutive cases. We aimed to include university hospitals as well as non-university hospitals to enhance generalizability of the findings. Nine high-volume medical centres in Germany, Austria and Switzerland with experience in SE-treatment were recruited: a) Germany: Epilepsy Centre Hessen/University of Marburg (F. Rosenow), University Hospital Kiel (N. Lang), Klinikum Osnabrück (C. Kellinghaus), Krankenhaus Barmherzige Brüder, Regensburg (C. Tilz); b) Austria: Christian-Doppler-Klinik Universitätsklinikum der Paracelsus Medizinischen Universität Salzburg, (E. Trinka), Krankenhaus Barmherzige Brüder, Linz (C. Tilz), University Hospital, Innsbruck (I. Unterberger); c) Switzerland: University Hospitals Basel (S. Rüegg), University Hospital Lausanne (A.O. Rossetti). We aimed to include university hospitals as well as non-university hospitals to enhance generalizability of the findings.

Personnel collecting data from the patient charts and entering data into the database were board certified neurologists, or neurology residents, or medical students in their final year, or study nurses, who were specifically instructed prior to data collection and personally supervised by one of the authors during the data collection and entry. Patients were treated in highly equipped emergency departments or intensive care units with electronical data entry and managing; thus, type, dosages and time points of drug administration could be easily ascertained. The increasing use of continuous Video-EEG monitoring also allowed for exact and reliable determination of cessation of NCSE, and in some cases also of NCSE onset.

### Ethical approval and funding

The local ethics committee of each participating center approved the final study protocol. The following ethics committees reviewed and approved the study: University Hospital of Marburg (Lead opinion for the German Institutions), University Hospital of Kiel, Chamber of Physicians Niedersachsen (for Osnabrück), Chamber of Physicians Bavaria (for Regensburg), Paracelsus University of Salzburg, Krankenhaus Barmherzige Brüder Linz, University Hospital of Innsbruck, University Hospital of Basel and University Hospital of Lausanne. Informed consent was waived owing to the purely observational character and complete anonymisation of the patients by all ethic committees except one (Innsbruck), for which IC was obtained for all patients from this centre. The study was registered at the German registry for clinical studies (DRKS) fulfilling the WHO-standards (DRKS00000725). The study was not funded by any governmental, research or commercial entity. Resources were drawn from non-specific research funds of each institution.

### Objectives

The main objective of the study is to document patient characteristics, treatment modalities and in-house outcome of adults treated for SE, and identify predictors of outcome. The data collected in this study could also identify gaps and opportunities for the management of this medical emergency. Lacking adequate prospective controlled trials, this will help both decision-making in clinical practice and designing future clinical trials.

Particularly, following questions will be addressed: 1) which factors determine successful SE treatment at each treatment step? 2) what are the complications associated with each substance and treatment modality? 3) which factors determine global outcome at the time of hospital discharge? (4) what is the relative prevalence of the semiological subgroups of SE? 5) is there a significant difference regarding SE treatment according to countries or institutions?

### Study population

All consecutive adult patients who are diagnosed with SE at admission or at any point of the in-patient treatment could be entered into the registry. SE is defined as a) seizures lasting >5 min, or b) recurrent seizures without regaining recovery of awareness over >5 min. or c) in comatose patients we applied the following EEG criteria [24] for non convulsive SE (NCSE): (A) Epileptiform discharges (EDs) > 2.5 Hz, or (B) EDs ≤ 2.5 Hz or rhythmic delta/theta activity (>0.5 Hz) AND one of the following: (i) EEG and clinical improvement after IV AED*, or (ii) subtle clinical ictal phenomena, or (iii) typical spatiotemporal evolution of the EEG pattern.

### Definition of inclusion and exclusion criteria

There is no commonly accepted time criterion for inclusion in SE trials. However, the recent large trials focusing on emergency first line therapy used seizure duration of 5 min or more as entry variable for convulsive SE [7, 8]. To increase comparability between our data and these studies, we chose the same operational definition. Other inclusion criteria were not defined.

Status-epilepticus-like EEG and clinical phenomena in hypoxic brain injury are considered pathophysiologically distinct from status epilepticus proper, and carry a very specific and generally poor prognosis [25]. Therefore, we excluded patients with status epilepticus-like encephalopathies after hypoxic brain injury. No other exclusion criteria were applied besides age (<16 years).

### Clinical data

The following variables were considered as crucial for an observational registry:

- demographics: age at admission, gender
- health variables at time of SE onset: modified Rankin scale, relevant known health problems (comorbidities), history of previous seizures, anticonvulsant treatment
- SE-related variables: Date and time of onset, semiology (worst seizure type), consciousness before treatment, etiology, EEG during SE (if applicable). These data will allow calculating the Status Epilepticus Severity Score (STESS, [26])
- Treatment-related variables: date and time of first treatment, treatment before admission to hospital, date and time of the first administration of any anticonvulsant substance used, bolus dose, bolus mode of administration, bolus infusion speed (if applicable), maximum daily dose and mode of administration, date and time of last dose applied, reason for discarding the substance, intubation date and time (if applicable), reason for intubation (airway protection vs. intentional coma induction for SE treatment), maximum serum levels of the substances in use (if applicable), adverse event documentation.
- outcome-related variables: date and time of SE cessation (if applicable), date and time of EEG-documentation of SE cessation (if applicable), day of discharge from hospital, modified Rankin scale at discharge, anticonvulsant medication at discharge (if applicable).

The modified Rankin-scale – a global outcome measure of morbidity and impairment - was chosen because of its easy applicability in a large variety of clinical scenarios with neurological diseases.

### Management protocol

No common management protocol will be imposed on the participating centres. However, most institutions have established an in-house protocol that is closely related to the national diagnosis and treatment guidelines published by the neurological societies of Germany, Austria and Switzerland [27].

In general, diagnosis of SE and related conditions is made using clinical examination, EEG or both. In most cases, emergency cranial imaging with CT or MRI as well as routine emergency blood analysis, sometimes lumbar puncture, is performed. Most frequently, the first treatment step consists in application of a benzodiazepine. If this is not successful, intravenous application of anticonvulsants such as (fos-)phenytoin, valproate, levetiracetam, or lacosamide follow. As third step, another anticonvulsant is added and/or therapeutic coma is initiated using propofol, midazolam or barbiturates. All treatment decisions will remain at the discretion of the attending physicians.

### Data quality

Data are collected prospectively from the admission of the patient to the discharge. Documentation has to be started latestat the third day after treatment to guarantee the data quality. Data collection is performed using a 4-page paper CRF (case report file) version or an eCRF through a registry website using a centre-specific login and password, and fully anonymized. At the beginning, each investigator has been trained in the usage of the CRF and eCRF.

### Sample size and data analysis

According to single centre observational registries [28–30] each treatment step carries a likelihood of between 40 and 60 % to represent the last treatment step before SE cessation. Thus, a cohort of approximately 1000 patients would be needed to reach a group size of 200–300 patients in the refractory group (i.e., resistant to first- and second-line treatments). This group size would be needed to assess approximately 20 possible predictors of outcome with a two-sided significance level of 5 %, a power of 80 % and an anticipated effect size of 10 %.

### Data analysis

Calculations will be conducted according to incident patients (especially for mortality, where each patient cannot count for several exposures), and episodes (for example, regarding treatment impact, where several exposures of the same patient are potentially informative). Interval-scaled and ordinal-scaled data will be presented as median/percentiles in addition to mean/standard deviation because normal distribution and variance homogeneity cannot easily be assumed for most of the variables. Categorical variables will be presented as frequency/percentages. Differences between outcome categories will be analyzed using non-parametric tests such as Mann–Whitney-*U*-test or Kruskal-Wallis-Test. Categorical variables will be tested using the Chi-square test or Fisher’s exact test (2x2 tables), as needed. Multivariable approaches will assess significance of variables of interest after multivariable analyses. *P* values of <0.05 will be considered as significant (two-sided testing). Statistical analysis will be performed using the latest version of SPSS (Chicago, Illinois)

## Discussion

When a large randomised controlled trial is not feasible, the second best option for evaluating SE treatment options is a prospective registry with a sufficient number of cases recruited from a sufficient number of institutions of different types (academic tertiary care centers and community hospitals). Ideally, a prospective registry should include many different sites in different countries. However, the coordination would require considerable effort, starting with organization and costs of meetings. In addition, a larger number of patients would be required to account for larger inhomogeneity between centers and countries, thus requiring larger resources for data acquisition and data analysis. Our efforts for acquiring additional funding (e.g. from drug companies or independent research organisations) showed that besides some general research fund resources from most of the centers, no further support would be available. In addition, a purely observational design avoids the ethical, legal and financial problems mentioned above while guaranteeing a reasonable degree of comparability and validity of the data. We feel that there is an urgent need for new data regarding the treatment options for established and refractory SE. Therefore, and for other reasons mentioned above, a prospective registry seems to represent the only solution to collect data in a meaningful time to bridge the latency until the results of a randomized trial could be available

At the same time, data from this registry can serve as a pilot database to generate and adapt hypotheses and determinants such as dose per body weight and infusion speed for any future trial.

To avoid the investigation of a restricted group of patients, we will include all adults with SE regardless of age, etiology and comorbidity except for patients with hypoxic brain damage. The individual factors influencing treatment success and outcome, particularly the reasons for failure and success of a substance in specific circumstances, are not well understood. Failure to include certain patient groups might result in a significant loss of generalizability. Owing to the minimum of exclusion and inclusion criteria and the simplicity of the observational flow, the registry is easy to implement in Non-University hospitals with limited staff and will most likely result in a patient mix representative of clinical practice. We will not include children because etiologies, treatment options and outcomes are markedly different. In our view, pediatric SE would need a data collection and analysis of its own, as well as postanoxic SE.

The evaluation of a large, heterogeneous patient group using standardized definitions and documentation will hopefully help in clinical decision making in an area of emergency medicine where data with higher level of evidence are still lacking.

## Abbreviations

SENSE: Sustained effort network for treatment of status epilepticus
SE: Status epilepticus
ILAE: International league against epilepsy
IC: Informed consent
EEG: Electroencephalography
STESS: Status epilepticus severity score
CT: Computer tomography
MRI: Magnetic resonance imaging
CRF: Case report file
